# Dichloridobis(2-dimethyl­amino-1,10-phenanthroline)cadmium(II)

**DOI:** 10.1107/S1600536809032358

**Published:** 2009-08-29

**Authors:** Hong Liang Li

**Affiliations:** a Department of Chemistry, Dezhou University, Dezhou 253023, People’s Republic of China

## Abstract

In the title complex, [CdCl_2_(C_14_H_13_N_3_)_2_], the Cd^II^ ion lies on a twofold rotation axis and assumes a distorted octa­hedral CdN_4_Cl_2_ coordination geometry. There is a π–π stacking inter­action between the symmetry-related 1,10-phenanthroline ligands with a centroid–centroid distance of 3.5578 (16) Å and a perpendicular distance of 3.445 (su?) Å between the relevant rings.

## Related literature

For background to the use of 1,10-phenanthroline derivatives in coordination chemistry, see: Liu *et al.* (2008 ▶). For a related structure, see: Zhang *et al.* (2008 ▶).
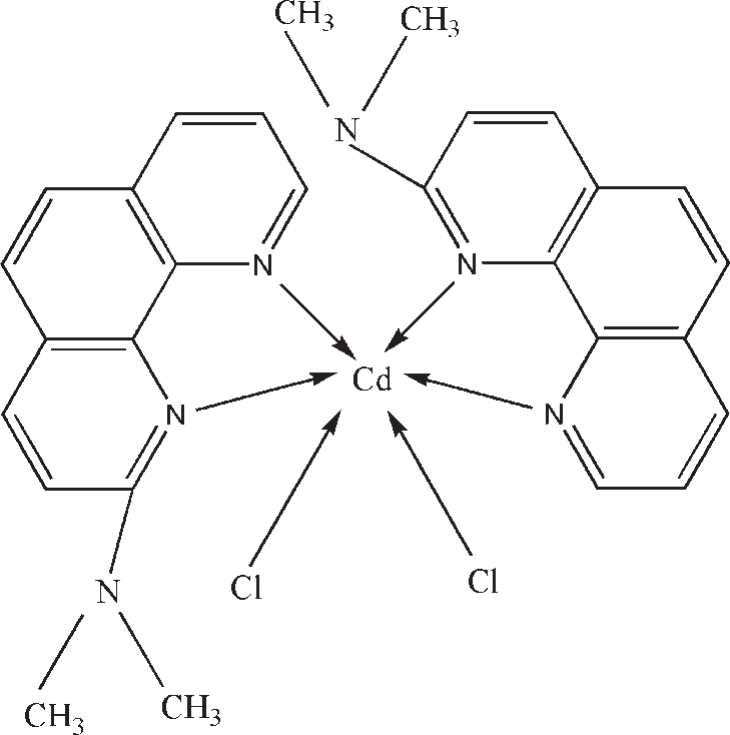


## Experimental

### 

#### Crystal data

[CdCl_2_(C_14_H_13_N_3_)_2_]
*M* *_r_* = 629.85
Monoclinic, 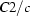

*a* = 17.161 (2) Å
*b* = 9.3572 (12) Å
*c* = 16.805 (2) Å
β = 110.343 (2)°
*V* = 2530.2 (6) Å^3^
*Z* = 4
Mo *K*α radiation
μ = 1.11 mm^−1^
*T* = 298 K
0.36 × 0.19 × 0.17 mm

#### Data collection

Bruker SMART APEX CCD diffractometer
Absorption correction: multi-scan (*SADABS*; Bruker, 1997 ▶) *T*_min_ = 0.692, *T*_max_ = 0.834
7147 measured reflections
2741 independent reflections
2512 reflections with *I* > 2σ(*I*)
*R*_int_ = 0.026

#### Refinement

*R*[*F* ^2^ > 2σ(*F* ^2^)] = 0.032
*wR*(*F* ^2^) = 0.078
*S* = 1.04
2741 reflections
170 parameters
H-atom parameters constrained
Δρ_max_ = 0.67 e Å^−3^
Δρ_min_ = −0.32 e Å^−3^

### 

Data collection: *SMART* (Bruker, 1997 ▶); cell refinement: *SAINT* (Bruker, 1997 ▶); data reduction: *SAINT*; program(s) used to solve structure: *SHELXS97* (Sheldrick, 2008 ▶); program(s) used to refine structure: *SHELXL97* (Sheldrick, 2008 ▶); molecular graphics: *SHELXTL* (Sheldrick, 2008 ▶); software used to prepare material for publication: *SHELXTL* and local programs.

## Supplementary Material

Crystal structure: contains datablocks I, global. DOI: 10.1107/S1600536809032358/bt5029sup1.cif

Structure factors: contains datablocks I. DOI: 10.1107/S1600536809032358/bt5029Isup2.hkl

Additional supplementary materials:  crystallographic information; 3D view; checkCIF report

## Figures and Tables

**Fig. 1.**
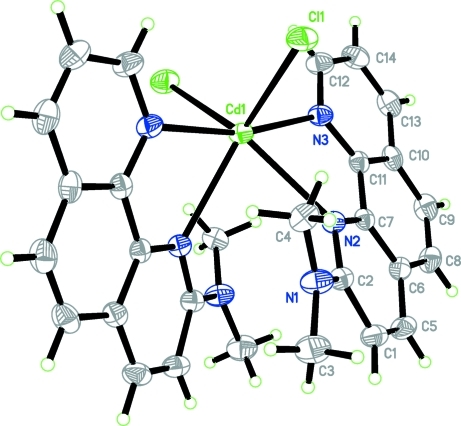
Coordination diagram of title complex with atom-numbering scheme for only asymmetric unit, and displacement ellipsoids being at the 30% probability level

## supplementary crystallographic information

### Comment

Derivatives of 1,10-phenanthroline play an important role in modern coordination chemistry (Liu *et al.*, 2008), and only one complex with 2-(dimethyl)amine-1,10-phenanthroline as ligand has been published (Zhang *et al.*, 2008) so far. Now   the crystal structure of title complex, which is the second complex dealing with 2-(dimethyl)amine-1,10-phenanthroline as ligand, is reported.

Fig. 1 and Table 1 show the coordination structure, with the Cd centre located on a crystallographic twofold axis. It is in a distorted octahedral geometry. There is a π-π stacking interaction involving symmetry related 1,10-phenanthroline ligands, with the relevant distances being *Cg*1···*Cg*2^i^ = 3.5578 (16) Å and *Cg*1···*Cg*2^i^_perp_ = 3.445 Å and α = 3.82° [symmetry code: (i) 1-*X*, -*Y*, -*Z*; *Cg*1 and *Cg*2 are the centroids of C1C2C5-C7/N2 ring and C6—C11 ring, respectively; *Cg*1···*Cg*2^1^_perp_ is the perpendicular distance from ring *Cg*1 to ring *Cg*2^i^; α is the dihedral between ring plane *Cg*1 and ring plane *Cg*2^i^].

### Experimental

10 ml me thanol solution of 2-(dimethyl)amine-1,10-phenanthroline (0.1438 g, 0.644 mmol) was added into 5 ml H_2_O solution of cadmium chloride (0.1485 g, 0.650 mmol), and the mixed solution was stirred for a few minutes. The yellow single crystals were obtained after the filtrate had been allowed to stand at room temperature for two weeks.

### Refinement

All H atoms were placed in calculated positions and refined as riding with C—H = 0.96 Å and *U*_iso_(H) = 1.5*U*_eq_(C) for methyl groups, and C—H = 0.93 Å and *U*_iso_(H) = 1.2*U*_eq_(C) for other H atoms.

### Crystal data

**Table:**

[CdCl_2_(C_14_H_13_N_3_)_2_]	*F*(000) = 1272
*M**_r_* = 629.85	*D*_x_ = 1.653 Mg m^−^^3^
Monoclinic, *C*2/*c*	Mo *K*α radiation, λ = 0.71073 Å
Hall symbol: -C 2yc	Cell parameters from 3730 reflections
*a* = 17.161 (2) Å	θ = 2.5–27.9°
*b* = 9.3572 (12) Å	µ = 1.11 mm^−^^1^
*c* = 16.805 (2) Å	*T* = 298 K
β = 110.343 (2)°	Block, yellow
*V* = 2530.2 (6) Å^3^	0.36 × 0.19 × 0.17 mm
*Z* = 4	

### Data collection

**Table:**

Bruker SMART APEX CCD diffractometer	2741 independent reflections
Radiation source: fine-focus sealed tube	2512 reflections with *I* > 2σ(*I*)
graphite	*R*_int_ = 0.026
φ and ω scans	θ_max_ = 27.0°, θ_min_ = 2.5°
Absorption correction: multi-scan (*SADABS*; Bruker, 1997)	*h* = −21→21
*T*_min_ = 0.692, *T*_max_ = 0.834	*k* = −6→11
7147 measured reflections	*l* = −21→21

### Refinement

**Table:**

Refinement on *F*^2^	Primary atom site location: structure-invariant direct methods
Least-squares matrix: full	Secondary atom site location: difference Fourier map
*R*[*F*^2^ > 2σ(*F*^2^)] = 0.032	Hydrogen site location: inferred from neighbouring sites
*wR*(*F*^2^) = 0.078	H-atom parameters constrained
*S* = 1.04	*w* = 1/[σ^2^(*F*_o_^2^) + (0.0465*P*)^2^ + 0.8626*P*] where *P* = (*F*_o_^2^ + 2*F*_c_^2^)/3
2741 reflections	(Δ/σ)_max_ = 0.002
170 parameters	Δρ_max_ = 0.67 e Å^−^^3^
0 restraints	Δρ_min_ = −0.32 e Å^−^^3^

### Special details

**Table:**

Geometry. All e.s.d.'s (except the e.s.d. in the dihedral angle between two l.s. planes) are estimated using the full covariance matrix. The cell e.s.d.'s are taken into account individually in the estimation of e.s.d.'s in distances, angles and torsion angles; correlations between e.s.d.'s in cell parameters are only used when they are defined by crystal symmetry. An approximate (isotropic) treatment of cell e.s.d.'s is used for estimating e.s.d.'s involving l.s. planes.
Refinement. Refinement of *F*^2^ against ALL reflections. The weighted *R*-factor *wR* and goodness of fit *S* are based on *F*^2^, conventional *R*-factors *R* are based on *F*, with *F* set to zero for negative *F*^2^. The threshold expression of *F*^2^ > σ(*F*^2^) is used only for calculating *R*-factors(gt) *etc*. and is not relevant to the choice of reflections for refinement. *R*-factors based on *F*^2^ are statistically about twice as large as those based on *F*, and *R*- factors based on ALL data will be even larger.

### Fractional atomic coordinates and isotropic or equivalent isotropic displacement parameters (Å^2^)

**Table:**

	*x*	*y*	*z*	*U*_iso_*/*U*_eq_	
C1	0.53071 (18)	−0.2662 (3)	0.14483 (19)	0.0453 (6)	
H1	0.5561	−0.3519	0.1680	0.054*	
C2	0.55652 (14)	−0.1339 (3)	0.18970 (16)	0.0366 (5)	
C3	0.6672 (2)	−0.2607 (3)	0.3007 (2)	0.0597 (8)	
H3A	0.6275	−0.3371	0.2899	0.090*	
H3B	0.6916	−0.2451	0.3608	0.090*	
H3C	0.7099	−0.2855	0.2785	0.090*	
C4	0.67199 (15)	−0.0021 (3)	0.29048 (17)	0.0442 (6)	
H4A	0.6495	0.0734	0.2503	0.066*	
H4B	0.7293	−0.0169	0.2972	0.066*	
H4C	0.6679	0.0237	0.3442	0.066*	
C5	0.46892 (18)	−0.2641 (3)	0.0683 (2)	0.0468 (6)	
H5	0.4529	−0.3484	0.0377	0.056*	
C6	0.42857 (14)	−0.1347 (3)	0.03479 (15)	0.0378 (5)	
C7	0.45301 (13)	−0.0134 (2)	0.08600 (14)	0.0324 (5)	
C8	0.36608 (16)	−0.1257 (3)	−0.04727 (16)	0.0483 (7)	
H8	0.3509	−0.2077	−0.0804	0.058*	
C9	0.32884 (17)	−0.0021 (3)	−0.07762 (17)	0.0507 (7)	
H9	0.2884	0.0010	−0.1315	0.061*	
C10	0.35021 (14)	0.1243 (3)	−0.02876 (15)	0.0410 (6)	
C11	0.40983 (14)	0.1189 (3)	0.05419 (14)	0.0346 (5)	
C12	0.39202 (17)	0.3585 (3)	0.07410 (18)	0.0488 (6)	
H12	0.4049	0.4381	0.1094	0.059*	
C13	0.31509 (18)	0.2587 (4)	−0.05904 (18)	0.0499 (7)	
H13	0.2766	0.2671	−0.1137	0.060*	
C14	0.33721 (18)	0.3755 (3)	−0.00872 (18)	0.0531 (7)	
H14	0.3163	0.4652	−0.0290	0.064*	
Cd1	0.5000	0.20663 (2)	0.2500	0.03387 (10)	
Cl1	0.61434 (4)	0.36432 (7)	0.22647 (5)	0.05136 (18)	
N1	0.62589 (13)	−0.1317 (2)	0.26011 (15)	0.0465 (5)	
N2	0.51504 (11)	−0.0126 (2)	0.16313 (12)	0.0339 (4)	
N3	0.42685 (13)	0.2347 (2)	0.10524 (14)	0.0396 (5)	

### Atomic displacement parameters (Å^2^)

**Table:**

	*U*^11^	*U*^22^	*U*^33^	*U*^12^	*U*^13^	*U*^23^
C1	0.0450 (15)	0.0335 (13)	0.0594 (17)	0.0015 (11)	0.0208 (13)	0.0007 (12)
C2	0.0310 (11)	0.0353 (12)	0.0446 (13)	−0.0017 (9)	0.0144 (10)	−0.0014 (10)
C3	0.0533 (18)	0.0496 (16)	0.068 (2)	0.0181 (14)	0.0110 (16)	0.0104 (15)
C4	0.0343 (12)	0.0454 (14)	0.0472 (14)	0.0030 (11)	0.0069 (11)	−0.0062 (12)
C5	0.0462 (15)	0.0371 (13)	0.0596 (17)	−0.0105 (11)	0.0216 (14)	−0.0121 (12)
C6	0.0340 (12)	0.0406 (13)	0.0390 (12)	−0.0075 (10)	0.0130 (10)	−0.0056 (11)
C7	0.0285 (11)	0.0375 (12)	0.0322 (11)	−0.0063 (9)	0.0117 (9)	−0.0011 (9)
C8	0.0455 (14)	0.0565 (17)	0.0404 (14)	−0.0104 (13)	0.0118 (12)	−0.0146 (13)
C9	0.0451 (15)	0.0664 (19)	0.0334 (13)	−0.0081 (13)	0.0045 (11)	−0.0037 (13)
C10	0.0317 (12)	0.0576 (16)	0.0340 (12)	−0.0023 (11)	0.0117 (10)	0.0045 (12)
C11	0.0303 (11)	0.0406 (13)	0.0340 (11)	−0.0070 (10)	0.0124 (9)	−0.0019 (10)
C12	0.0505 (15)	0.0382 (14)	0.0493 (15)	0.0040 (12)	0.0066 (12)	0.0062 (12)
C13	0.0396 (14)	0.0670 (18)	0.0368 (14)	0.0022 (13)	0.0053 (11)	0.0113 (13)
C14	0.0520 (16)	0.0485 (16)	0.0535 (16)	0.0080 (13)	0.0115 (13)	0.0168 (14)
Cd1	0.03261 (15)	0.02812 (14)	0.03351 (15)	0.000	0.00217 (10)	0.000
Cl1	0.0486 (4)	0.0394 (4)	0.0652 (4)	−0.0099 (3)	0.0188 (3)	−0.0034 (3)
N1	0.0360 (11)	0.0370 (12)	0.0562 (13)	0.0051 (9)	0.0029 (10)	0.0034 (10)
N2	0.0299 (9)	0.0336 (10)	0.0356 (10)	−0.0019 (8)	0.0080 (8)	−0.0006 (8)
N3	0.0395 (11)	0.0342 (10)	0.0406 (12)	0.0013 (9)	0.0083 (9)	0.0032 (9)

### Geometric parameters (Å, °)

**Table:**

C1—C5	1.353 (4)	C8—C9	1.334 (4)
C1—C2	1.437 (4)	C8—H8	0.9300
C1—H1	0.9300	C9—C10	1.413 (4)
C2—N2	1.332 (3)	C9—H9	0.9300
C2—N1	1.356 (3)	C10—C13	1.412 (4)
C3—N1	1.445 (4)	C10—C11	1.415 (3)
C3—H3A	0.9600	C11—N3	1.349 (3)
C3—H3B	0.9600	C12—N3	1.325 (3)
C3—H3C	0.9600	C12—C14	1.392 (4)
C4—N1	1.441 (3)	C12—H12	0.9300
C4—H4A	0.9600	C13—C14	1.353 (4)
C4—H4B	0.9600	C13—H13	0.9300
C4—H4C	0.9600	C14—H14	0.9300
C5—C6	1.411 (4)	Cd1—N3^i^	2.332 (2)
C5—H5	0.9300	Cd1—N3	2.332 (2)
C6—C7	1.398 (3)	Cd1—N2^i^	2.582 (2)
C6—C8	1.425 (3)	Cd1—N2	2.582 (2)
C7—N2	1.362 (3)	Cd1—Cl1	2.5928 (7)
C7—C11	1.446 (3)	Cd1—Cl1^i^	2.5928 (7)
			
C5—C1—C2	118.9 (3)	N3—C11—C10	121.4 (2)
C5—C1—H1	120.6	N3—C11—C7	118.8 (2)
C2—C1—H1	120.6	C10—C11—C7	119.8 (2)
N2—C2—N1	118.9 (2)	N3—C12—C14	123.4 (3)
N2—C2—C1	121.8 (2)	N3—C12—H12	118.3
N1—C2—C1	119.2 (2)	C14—C12—H12	118.3
N1—C3—H3A	109.5	C14—C13—C10	120.2 (2)
N1—C3—H3B	109.5	C14—C13—H13	119.9
H3A—C3—H3B	109.5	C10—C13—H13	119.9
N1—C3—H3C	109.5	C13—C14—C12	118.6 (3)
H3A—C3—H3C	109.5	C13—C14—H14	120.7
H3B—C3—H3C	109.5	C12—C14—H14	120.7
N1—C4—H4A	109.5	N3^i^—Cd1—N3	167.08 (10)
N1—C4—H4B	109.5	N3^i^—Cd1—N2^i^	67.85 (7)
H4A—C4—H4B	109.5	N3—Cd1—N2^i^	123.76 (7)
N1—C4—H4C	109.5	N3^i^—Cd1—N2	123.76 (7)
H4A—C4—H4C	109.5	N3—Cd1—N2	67.85 (7)
H4B—C4—H4C	109.5	N2^i^—Cd1—N2	74.81 (9)
C1—C5—C6	120.4 (3)	N3^i^—Cd1—Cl1	86.19 (6)
C1—C5—H5	119.8	N3—Cd1—Cl1	86.47 (6)
C6—C5—H5	119.8	N2^i^—Cd1—Cl1	140.17 (4)
C7—C6—C5	116.8 (2)	N2—Cd1—Cl1	97.82 (5)
C7—C6—C8	120.8 (2)	N3^i^—Cd1—Cl1^i^	86.47 (6)
C5—C6—C8	122.5 (2)	N3—Cd1—Cl1^i^	86.19 (6)
N2—C7—C6	123.9 (2)	N2^i^—Cd1—Cl1^i^	97.82 (5)
N2—C7—C11	118.5 (2)	N2—Cd1—Cl1^i^	140.17 (4)
C6—C7—C11	117.5 (2)	Cl1—Cd1—Cl1^i^	110.63 (3)
C9—C8—C6	121.4 (3)	C2—N1—C4	122.0 (2)
C9—C8—H8	119.3	C2—N1—C3	122.5 (2)
C6—C8—H8	119.3	C4—N1—C3	114.6 (2)
C8—C9—C10	120.7 (2)	C2—N2—C7	117.7 (2)
C8—C9—H9	119.7	C2—N2—Cd1	129.38 (16)
C10—C9—H9	119.7	C7—N2—Cd1	110.11 (14)
C13—C10—C11	117.3 (3)	C12—N3—C11	118.9 (2)
C13—C10—C9	123.0 (2)	C12—N3—Cd1	121.04 (18)
C11—C10—C9	119.7 (2)	C11—N3—Cd1	119.46 (16)
			
C5—C1—C2—N2	8.2 (4)	N1—C2—N2—Cd1	−30.5 (3)
C5—C1—C2—N1	−170.5 (3)	C1—C2—N2—Cd1	150.79 (19)
C2—C1—C5—C6	−2.3 (4)	C6—C7—N2—C2	2.4 (3)
C1—C5—C6—C7	−2.9 (4)	C11—C7—N2—C2	−177.1 (2)
C1—C5—C6—C8	176.8 (3)	C6—C7—N2—Cd1	−160.33 (18)
C5—C6—C7—N2	3.1 (3)	C11—C7—N2—Cd1	20.2 (2)
C8—C6—C7—N2	−176.6 (2)	N3^i^—Cd1—N2—C2	7.3 (2)
C5—C6—C7—C11	−177.4 (2)	N3—Cd1—N2—C2	−179.2 (2)
C8—C6—C7—C11	2.9 (3)	N2^i^—Cd1—N2—C2	−42.08 (16)
C7—C6—C8—C9	−0.3 (4)	Cl1—Cd1—N2—C2	97.91 (18)
C5—C6—C8—C9	−180.0 (3)	Cl1^i^—Cd1—N2—C2	−126.03 (17)
C6—C8—C9—C10	−0.3 (4)	N3^i^—Cd1—N2—C7	167.33 (14)
C8—C9—C10—C13	177.5 (3)	N3—Cd1—N2—C7	−19.10 (14)
C8—C9—C10—C11	−2.0 (4)	N2^i^—Cd1—N2—C7	117.98 (17)
C13—C10—C11—N3	6.0 (3)	Cl1—Cd1—N2—C7	−102.02 (14)
C9—C10—C11—N3	−174.5 (2)	Cl1^i^—Cd1—N2—C7	34.03 (18)
C13—C10—C11—C7	−174.8 (2)	C14—C12—N3—C11	1.1 (4)
C9—C10—C11—C7	4.7 (4)	C14—C12—N3—Cd1	−169.7 (2)
N2—C7—C11—N3	−6.3 (3)	C10—C11—N3—C12	−5.8 (4)
C6—C7—C11—N3	174.1 (2)	C7—C11—N3—C12	175.0 (2)
N2—C7—C11—C10	174.5 (2)	C10—C11—N3—Cd1	165.14 (17)
C6—C7—C11—C10	−5.1 (3)	C7—C11—N3—Cd1	−14.1 (3)
C11—C10—C13—C14	−1.5 (4)	N3^i^—Cd1—N3—C12	−16.5 (2)
C9—C10—C13—C14	179.0 (3)	N2^i^—Cd1—N3—C12	135.9 (2)
C10—C13—C14—C12	−2.9 (4)	N2—Cd1—N3—C12	−171.9 (2)
N3—C12—C14—C13	3.2 (5)	Cl1—Cd1—N3—C12	−72.0 (2)
N2—C2—N1—C4	−21.3 (4)	Cl1^i^—Cd1—N3—C12	39.0 (2)
C1—C2—N1—C4	157.4 (2)	N3^i^—Cd1—N3—C11	172.75 (18)
N2—C2—N1—C3	170.2 (3)	N2^i^—Cd1—N3—C11	−34.9 (2)
C1—C2—N1—C3	−11.0 (4)	N2—Cd1—N3—C11	17.35 (17)
N1—C2—N2—C7	170.7 (2)	Cl1—Cd1—N3—C11	117.28 (18)
C1—C2—N2—C7	−8.0 (3)	Cl1^i^—Cd1—N3—C11	−131.75 (18)

Symmetry codes: (i) −*x*+1, *y*, −*z*+1/2.
